# Dendrites differ from axons in patterns of microtubule stability and polymerization during development

**DOI:** 10.1186/1749-8104-4-26

**Published:** 2009-07-14

**Authors:** Katherine M Kollins, Robert L Bell, Matthew Butts, Ginger S Withers

**Affiliations:** 1 Department of Biology, Boyer Ave, Whitman College, Walla Walla, WA 99362, USA; 2 Center for Research on Occupational and Environmental Toxicology, Oregon Health and Science University, SW Sam Jackson Park Road, Portland, OR 97239, USA; 3 Department of Neurobiology and Anatomy, Drexel University College of Medicine, Queen Lane, Philadelphia, PA 19129, USA

## Abstract

**Background:**

Dendrites differ from axons in patterns of growth and development, as well as in morphology. Given that microtubules are key structural elements in cells, we assessed patterns of microtubule stability and polymerization during hippocampal neuron development *in vitro *to determine if these aspects of microtubule organization could distinguish axons from dendrites.

**Results:**

Quantitative ratiometric immunocytochemistry identified significant differences in microtubule stability between axons and dendrites. Most notably, regardless of developmental stage, there were high levels of dynamic microtubules throughout the dendritic arbor, whereas dynamic microtubules were predominantly concentrated in the distal end of axons. Analysis of microtubule polymerization using green fluorescent protein-tagged EB1 showed both developmental and regional differences in microtubule polymerization between axons and dendrites. Early in development (for example, 1 to 2 days *in vitro*), polymerization events were distributed equally in both the anterograde and retrograde directions throughout the length of both axons and dendrites. As development progressed, however, polymerization became biased, with a greater number of polymerization events in distal than in proximal and middle regions. While polymerization occurred almost exclusively in the anterograde direction for axons, both anterograde and retrograde polymerization was observed in dendrites. This is in agreement with predicted differences in microtubule polarity within these compartments, although fewer retrograde events were observed in dendrites than expected.

**Conclusion:**

Both immunocytochemical and live imaging analyses showed that newly formed microtubules predominated at the distal end of axons and dendrites, suggesting a common mechanism that incorporates increased microtubule polymerization at growing process tips. Dendrites had more immature, dynamic microtubules throughout the entire arbor than did axons, however. Identifying these differences in microtubule stability and polymerization is a necessary first step toward understanding how they are developmentally regulated, and may reveal novel mechanisms underlying neuron maturation and dendritic plasticity that extend beyond the initial specification of polarity.

## Background

A fundamental problem in cell biology is how morphologically and functionally distinct compartments are assembled and maintained in polarized cells. For hippocampal neurons developing *in vitro*, polarization occurs as structurally equivalent immature minor processes differentiate into mature axonal and dendritic arbors [1,2]. During this developmental progression, dendrites diverge from axons through a stereotypic sequence of morphogenesis, suggesting that at some level the cytoskeleton is being organized differently [3]. In fact, evidence suggests that local control of microtubule polymerization [4,5] and stability [6] both play a role in the initial specification of the axon and in minor process outgrowth [7,8]. Thus, it may be that differences in local regulation of microtubule polymerization or stability also underlie the faster rate of growth observed in axons [2,9,10], as well as contribute to the delayed formation and maturation of the dendritic arbor [3,11,12].

Previous work has shown that net axon growth involves both dynein motor-driven transport of short microtubules [13,14] and the formation of microtubule polymers within the axonal growth cone [5,15-17]. Correspondingly, molecular markers for newly assembled microtubules are concentrated at growing ends, while markers for more mature microtubules are found in the stable proximal and middle regions of axons [18-21]. Few studies have tested whether dendritic growth depends on tubulin subunit addition to the same extent, but overexpression of cypin, a protein that promotes microtubule polymerization, has been shown to increase the size of the dendritic arbor [22,23].

One characteristic difference that arises during maturation of the axon and dendrites is their intrinsic microtubule polarity. Previous electron microscopic analysis showed that axons of mature vertebrate neurons contained microtubules that were oriented exclusively with plus-ends projecting away from the soma, whereas the dendrites contained a combination of microtubules with mixed polarity [24]. Current imaging techniques allow microtubule polymerization to be examined directly in living cells, and can be used to determine how well this static view represents the dynamic nature of the microtubule network. Microtubule binding proteins that associate specifically with the distal plus-ends of actively polymerizing microtubules (called +TIPS), when tagged with green fluorescent protein (GFP), produce fluorescent 'comets' that track in the direction of polymerization [25,26]. Several *in vitro *studies have characterized GFP-+TIP dynamics, confirming that real-time localization of these proteins directly reflects the polarity of microtubule assembly in a variety of neuronal types [13,27-31]. One such protein, EB1, binds microtubules with high affinity and has been used in live imaging studies to study patterns of polymerization [32-35]. Thus, these fluorescent tools have the potential to reveal 'hot regions' of microtubule polymerization associated with active growth, as well as the orientation of microtubule polymerization, with the caveat that expression of a fusion protein can interfere with the function of the native protein.

The current study takes advantage of fluorescent imaging approaches to determine whether the spatial and temporal patterns of microtubule stability and polymerization differ between developing axons and dendrites of cultured hippocampal neurons. In this well-characterized model system, morphological stages of development can be observed readily [3]. In these cells, axons form first and grow rapidly. After the initial specification of the axon, development proceeds with an extended period of dendritic growth and maturation, during which dendrites become tapered, the arbor becomes highly branched, and postsynaptic specializations (for example, dendritic spines), appear. Quantitative immunofluorescence was used to compare the distribution of tyrosinated (Tyr) and acetylated (Ac) tubulin, post-translational modifications that reflect newly assembled (that is, dynamic) and mature stable microtubules, respectively [36,37]. Local patterns of microtubule polymerization were determined by measuring the distribution and directionality of fluorescent comets in living neurons expressing GFP-tagged EB1. Our analyses showed that axons and dendrites develop significant differences in both regional stability and ongoing polymerization of microtubules, each following distinct time courses. Dendrites could be distinguished from axons in patterns of microtubule stability within the first 2 days *in vitro *(DIV), whereas differences in patterns of polymerization arose later. Understanding how these differences are developmentally regulated may reveal novel mechanisms underlying neuron maturation and dendritic plasticity that extend beyond the initial specification of polarity.

## Materials and methods

### Hippocampal cultures

Primary neuron cultures were prepared from hippocampi of embryonic day 18 Sprague-Dawley rats (Taconic, Rensselaer, NY, USA) as previously described [38] except where noted below. Dissociated neurons were plated onto poly-L-lysine coated glass coverslips at approximately 15,000 cells/cm^2 ^for transfection experiments and at lower densities of 2,000 to 4,000 cells/cm^2 ^for immunocytochemistry studies, to facilitate visualization of separate neuronal processes, and maintained in Neurobasal medium (Gibco, Invitrogen, Carlsbad, CA, USA) with either B27 (Gibco, Invitrogen) or N2 supplements [39] and Glutamax (Gibco, Invitrogen). At 2 days post-plating, 5 μM cytosine β-D-arabinofuranoside (Sigma-Aldrich, St. Louis, MO, USA) was added to the co-cultures to prevent further glial cell division.

### PC12 cultures

PC12 rat pheochromocytoma cells (ATCC number: CRL-1721) were grown and treated with nerve growth factor (50 ng/ml; Sigma-Aldrich) to induce a neuron-like phenotype, as described previously [40,41]. After 7 to 10 days, differentiated PC12 cells were maintained in Dulbecco's modified Eagle's medium (DMEM) supplemented with 1% horse serum (HS) and 50 ng/ml nerve growth factor, which was refreshed by exchanging one-third of the plating volume every other day during the culture period.

### Antibodies and immunocytochemistry

To study the distribution of newly assembled and stable microtubules, monomeric tubulin was extracted by placing neuronal cultures in microtubule stabilizing buffer (80 mM PIPES pH 6.8, 1 mM MgCl_2_, 5 mM EGTA with 0.5% Triton X-100) for 30 s followed immediately by fixation in -20°C methanol for 3 to 5 minutes, and rehydration in 0.15 M Tris HCl, 0.15 M NaCl, and 0.1% Triton X-100. To ensure that there were no artifacts associated with methanol fixation, additional neurons were extracted by briefly rinsing in the microtubule stabilizing buffer PHEM (60 mM PIPES, 25 mM HEPES, 10 mM EGTA, 2 mM MgCl_2_, pH 6.9), followed by 0.5% Triton X-100 and 10 μm taxol in PHEM followed by fixation in 4% formaldehyde, 4% sucrose in phosphate-buffered saline (pH 7.4) [42]. Similar results were observed between these two extraction methods. Neurons were double-immunostained as described previously [43] using anti-acetylated tubulin (Sigma-Aldrich, clone 6–11B-1 mouse purified immunoglobulin, 1:1,000) and anti-tyrosinated tubulin (Chemicon, Temecula, CA, USA, clone YL1/2 rat monoclonal antibodies, 1:500). Total tubulin was assessed using anti-mouse alpha tubulin (DM1A, Sigma-Aldrich, 1:1,000), or anti-rabbit neuron-specific beta III tubulin (Covance Research Products, Princeton, NJ, USA). Secondary antibodies used were: Alexafluor goat anti-rabbit 546 (Molecular Probes, Invitrogen, Carlsbad, CA, USA; 1:500); FITC goat anti-rat (Jackson Immunoresearch Laboratories, West Grove, PA, USA; 1:500); and Alexafluor goat anti-mouse 488 (Molecular Probes; 1:400). All fixed and immunostained coverslips were rinsed, and mounted with Elvanol anti-fade medium (133.5 mM Tris HCl, 9.5 mM poly-vinyl alcohol, 45 mM Dabco, 3.6 mM glycerol).

### GFP-fusion constructs and transient transfection

The GFP-tagged EB1 plasmid (pEGFP-N1 vector, Clontech, Palo Alto, CA, USA) was obtained from Lynn Cassimeris, Lehigh University, Bethlehem, PA, USA [34]. Cells were transfected with 1.6 to 2 μg/ml purified DNA per 6-cm dish, in glial-conditioned medium. DOTAP (Roche Applied Science, Indianapolis, IN, USA) was used to transfect EB1-GFP into hippocampal neurons immediately after their isolation, and expression was examined between 1 and 17 DIV. In addition, Effectene (Qiagen, Valencia, CA, USA) and Lipofectamine 2000 (Gibco, Invitrogen) were used for transfection of hippocampal neurons performed between 3 and 10 DIV, and for PC12 cells, with EB1-GFP expression examined 24 to 48 h thereafter. For all lipid-mediated transfections, after a 4-h incubation in the transfection mixture at 37°C, neuronal coverslips were transferred to glial-feeder co-culture dishes and maintained in Neurobasal medium supplemented with 1 mM kynurenic acid (Sigma-Aldrich) to reduce excitotoxicity, and 2 mM sodium butyrate (Sigma-Aldrich) to enhance expression of cytomegalovirus (CMV) promoter-driven genes.

### Fluorescence microscopy and live cell imaging

Images of immunostained neurons were acquired using a Leica DM-RA2 epifluorescence microscope (Leica Microsystems, Bannockburn, IL, USA) with a high resolution motorized stage (ProScan, Prior Scientific, Rockland, MA, USA) coupled to a cooled CCD camera (CoolSnap fx 20 MHz Camera, Photometrics, Tucson, AZ, USA), using identical camera settings for fluorescent images (500 ms exposure). Cells were selected for acquisition using a forced sampling scheme. Briefly, the stage was moved at fixed intervals and digital images of the cells nearest the center of the field were acquired with Fluotar 20× and 40× objectives using MetaMorph software (MDS Analytical Technologies, Downingtown, PA, USA).

For live imaging of hippocampal neurons and PC12 cells, plated coverslips were sealed into a heated imaging chamber (Warner Instruments, Hamden, CT, USA) in phenol red-free minimal essential medium (MEM, Gibco, Invitrogen) supplemented with 30 μM glucose, 1 mM pyruvate, 500 μM kynurenic acid, and the antioxidants N-acetyl-cysteine (60 μM; Sigma-Aldrich), Trolox (20 μM; Sigma-Aldrich)) and Oxyrase (1:100; Oxyrase, Inc., Mansfield, OH, USA) to minimize phototoxicity and bleaching, and maintained at 34.5 to 36°C throughout the duration of the recording. Images were acquired with either a Fluotar 40× or Plan Apo 63×/1.32 NA objective using a Leica DM-RXA microscope (Leica Microsystems) coupled to a Princeton Instruments Micromax 5 Mhz CCD camera (Roper Scientific, Trenton, NJ, USA) or with a Leica DM-RA2 microscope with a Photometrics CoolSNAP fx 20 MHz camera. Time series stacks of images were acquired with MetaMorph software using either the 'stream acquire' (capturing continuous 600 ms exposures for durations of 30 to 60 s) or 'time series' function (capturing 750 ms or 1 s exposures for durations of 30 s to 2 minutes). During recording, a single plane of focus was maintained, which was sufficient to visualize EB1-GFP comets throughout the thickness of axons and most dendrites.

### Image processing and data analysis

Ratio imaging of immunostained neurons was performed using MetaMorph software. First, to control for nonuniformity in the image field, shading corrections and background subtraction were applied, using an image from a blank slide acquired under the same acquisition parameters. Next, to compare fluorescent intensity of the mid-shaft with the distal tip of dendrites, each dendrite was traced from cell body to tip to determine its length. A 5-μm segment from the midpoint, and a 5-μm segment from within the last 10 microns of the tip were selected (purposefully excluding the growth cone), and the 'line scan' function was used to obtain the mean pixel intensity for each of these regions for each antibody. The ratio of mid-shaft to distal region for both acetylated and tyrosinated forms of tubulin was calculated by dividing the value obtained at the mid-shaft region by the value obtained at the distal region. The same method was used to quantify immunofluorescence in axons.

**Figure 1:**
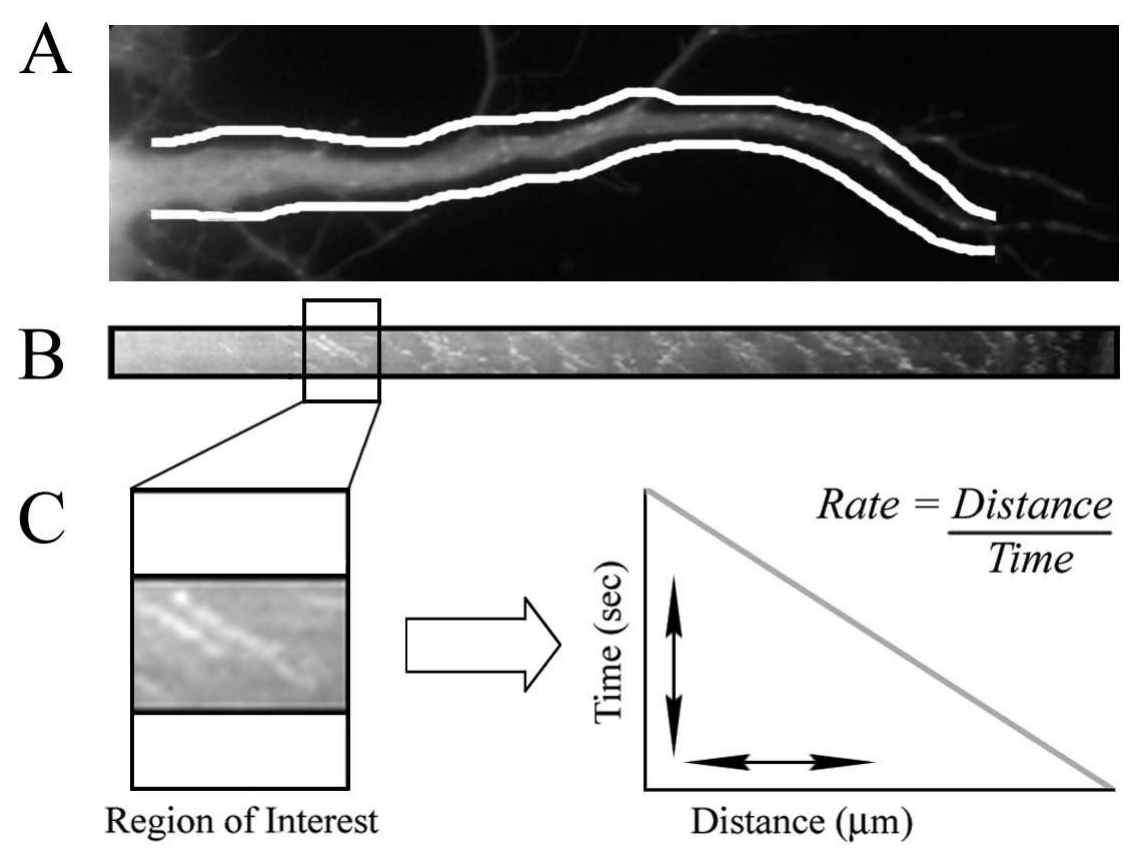
**Quantitative analysis of microtubule polymerization in axons and dendrites was performed using kymographs**. **(A) **Representative segment from the dendrite of a 2-DIV neuron expressing GFP-tagged EB1. White lines outline the region of the dendrite from which the kymograph was generated. In these analyses, the entire width of each axon or dendrite was sampled. **(B) **The corresponding kymograph detected multiple polymerization events in both the anterograde and retrograde directions. Proximal to distal neurite length is indicated on the y-axis, and time is represented on the x-axis. The kymographic trace shown here was compiled from 20 frames, acquired as a stream for a duration of 12 s. **(C) **Enlarged segment from the kymograph showing how comet slope was used to determine the rate and distance of individual polymerization events. See Additional file 1 to view the accompanying recording.

EB1-GFP comets in axons and dendrites were analyzed from digital image stacks using MetaMorph software. In 1-day-old cultures, only neurons that had developed a single axon that was at least 10 μm longer than the other minor processes of the cell were analyzed (in accordance with previous work that shows this length difference is predictive of the axon) [3]. In older cultures, the identity of axons and dendrites was based on standard morphological criteria and was unambiguous. Regions along axons and dendrites were categorized and grouped for analysis based on location with respect to the cell body or growth cone. For cells where the entire length of a process could be contained within the field of view, the process was divided into three equal regions: proximal, middle and distal. This was the case for cells at 1 to 2 DIV and for the dendrites at all ages. In older cells where the axon length was beyond the field of view, proximal was defined as within 50 μm of the soma, distal was within 50 μm of the growth cone, and the remainder of the axon was defined as the middle region. Only comets that could be detected in multiple, consecutive frames were selected for analysis. Individual EB1-GFP comet trajectories were determined using the 'kymograph' function of MetaMorph imaging software, which generates a cross-sectional view of pixel-intensity values throughout the stack of planes captured over time (Figure 1; Additional file 1). Briefly, lines were drawn along the entire length of the neuronal process, or the entire segment within the field of view. The line was then expanded to bracket the width of the process. Next, the kymograph function was used to find maximal pixel intensities at each point along the expanded cross-sectional transept for each plane in the image stack. These values were then plotted for each plane, with the time indicated on the x-axis and position along the neurite on the y-axis. Thus, moving comets, indicative of tubulin polymerization, appeared as diagonal lines whose slopes were a measure of the rate of polymerization and the direction of polymerization (anterograde, or toward the growth cone, versus retrograde, or toward the soma). Region statistics were determined for neuronal populations at each time point, including the number of polymerization events per unit length for both axons and dendrites, and the proportion of events that occurred in the anterograde versus retrograde direction. Individual events identified with the kymographs were corroborated manually by tracking movements frame by frame.

## Results

### Tyrosinated microtubules are abundant in distal ends of axons and tips of developing dendrites

**Figure 2:**
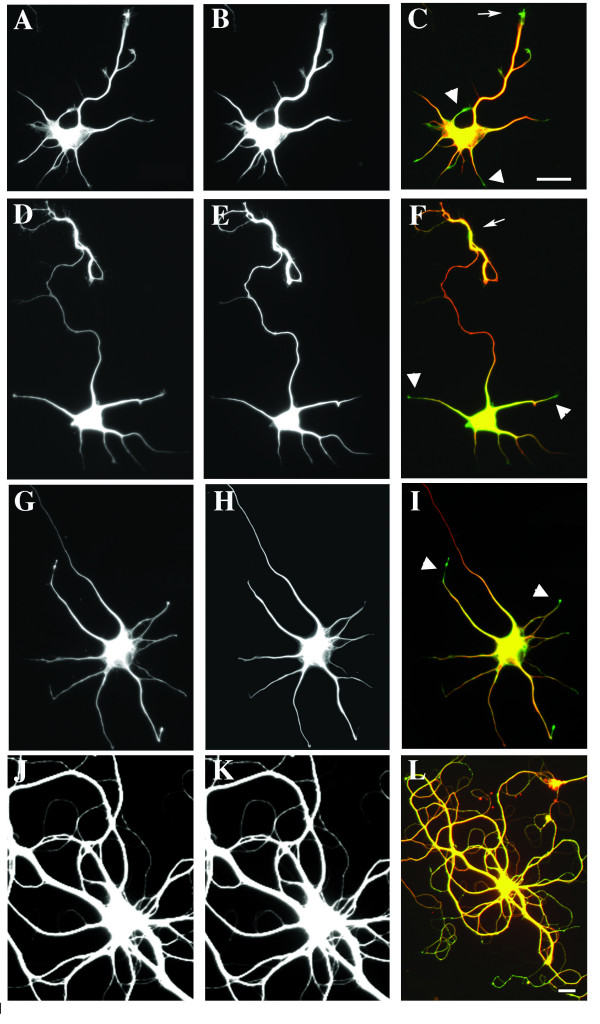
**Localization of dynamic and stable microtubules**. **(A-I) **Dynamic, tyrosinated (Tyr; left panels), and stable, acetylated (Ac; middle panels), microtubules and their overlap in merged images (Tyr, green; Ac, red; right panels) in cultured hippocampal neurons are shown at 2 (A-C), 4 (D-F), 7 (G-I) and 19 DIV (J-L). High levels of Tyr-tubulin immunofluorescence were apparent throughout the dendritic arbor in all stages examined. In addition, Tyr-tubulin was concentrated at growing tips of dendrites early in development (2 to 7 DIV, arrowheads) and distal axons at all stages stages where the ends could be observed in relative isolation (arrows). Tyr-tubulin immunofluorescence was decreased within the axonal shafts compared to Ac-tubulin, apparent as red regions in merged images. In contrast, dendrites appear yellow-green, suggesting higher proportions of Tyr-tubulin to Ac-tubulin throughout the dendritic arbor. Scale bars: 20 μm.

To assess spatial and temporal patterns of microtubule stability in axons and dendrites of developing hippocampal neurons, the distribution of dynamic and stable microtubules was determined using antibodies to tyrosinated (Tyr) and acetylated (Ac) tubulin, respectively (Figure 2). Key stages of dendritic development chosen for quantitative analysis were, first, minor process (that is, immature dendrite) growth following axon specification (2 DIV; Figure 2A–C), and second, differentiation of the dendritic arbor characterized by the development of taper and formation of branches (4 and 7 DIV; Figure 2D–F and 2G–I, respectively). A third late stage of dendrite maturation (19 DIV; Figure 2J–L) was examined qualitatively, but not quantitatively, because axon fasiculation along dendrites at this time-point prevented individual processes from being measured.

Within axons, Tyr-tubulin was enriched in growth cones (for example, note strong staining in axonal growth cones in Figure 2A; arrow in corresponding merged image, Figure 2C) as well as in the distal region of the axon (Figure 2D, F). Ac-tubulin was proportionally higher within the proximal and middle segments of the axon (Figure 2E, H; red in the corresponding merged images, Figure 2F, I). Similar patterns have been reported for axons of sympathetic neurons, suggesting common spatial regulation of microtubule stability among developing neuronal cell types [18-21].

During the first week in culture, dendritic growth cones stained intensely for Tyr-tubulin (Figure 2, arrowheads), similar to the distal Tyr-tubulin enrichment observed in axons. By 19 DIV, growth cones on dendrites could no longer be detected. At this late stage of development, growth cones could be present, though not apparent with immunostaining of the crowded neuronal field; alternatively, a loss of dendritic growth cones could reflect a change in the maturational state of the arbor.

**Figure 3:**
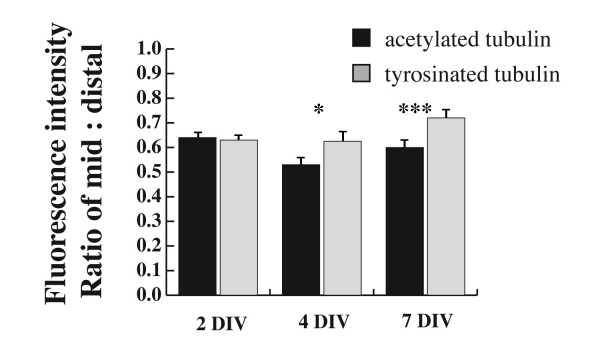
**Tyr-tubulin is increased in distal dendrites at stages of development when dendrite outgrowth is robust**. The relative amount of change between mid-shaft and distal regions of dendrites was determined for both Tyr-tubulin and Ac-tubulin by determining the ratio of fluorescence intensity between the two locations for each antibody. At 2 DIV, both Ac and Tyr show a similar distribution from mid- to distal shaft, but Tyr becomes significantly increased at 4 and 7 DIV. Values were determined by dividing the fluorescence intensity at the mid-shaft by that of the distal region. Tests of significance were done using a Student's *t*-test: 2 DIV, n = 122 dendrites; 4 DIV, n = 94 dendrites; 7 DIV, n = 59; *P < 0.05; ***P < 0.0001. Error bars indicate standard error of the mean.

Not every immature dendrite had a well-defined growth cone, but even those without showed Tyr-tubulin immunoreactivity. To determine whether the proportion of Tyr-tubulin was significantly increased over Ac-tubulin in distal dendrites, we determined the ratio of the fluorescence intensity for each antibody between two defined regions of the dendrite: the middle segment of the dendrite and 10 μm from the visible end of the tip, purposefully excluding the growth cone, which is typically motile and was clearly enriched in Tyr-tubulin compared to Ac-tubulin. In fact, the relative amount of Tyr-tubulin at the distal dendrite was significantly greater than that for Ac-tubulin at both 4 and 7 DIV (Figure 3). This time period coincides with a period of robust outgrowth of the dendritic arbor in cultured hippocampal neurons [3], and so increased levels of dynamic microtubules could contribute to that growth. No significant changes were detected at 2 DIV, but this is a time when there is little net extension of dendrites and so this finding also fits well with the prediction that dynamic microtubules are associated with outgrowth.

### Differential distribution of dynamic microtubules in shafts of dendrites and axons

Regardless of developmental time in culture, the entire dendritic arbor exhibited pronounced immunofluorescence for Tyr-tubulin (Figure 2A, D, G, J) as well as Ac-tubulin (Figure 2B, E, H, K), whereas Tyr-tubulin was clearly less abundant in the proximal and middle regions of axons. This suggested that the distribution of acetylated and tyrosinated microtubules along the dendrite shaft was more closely parallel than reciprocally proportional. To test this, we determined the ratio of immunofluorescence intensity of dynamic and stable microtubules at the midpoint and distal ends of the axon and dendrites of individual cells, and then compared the magnitude of difference (midshaft-to-distal). To control for potential variability in staining intensity between cells, this analysis was limited to cells at 2 DIV, where both the entire axon and dendritic field were contained within a single digital micrograph. With this comparison, the mean ratio of Ac-tubulin to Tyr-tubulin was 40% lower in dendrites than in axons (mean value of ratio Ac:Tyr ± standard error: dendrites, 4.68 ± 0.3; axons, 7.23 ± 1.79). Thus, there was proportionally more dynamic tubulin within dendrite shafts than within axons at this developmental time-point.

**Figure 4:**
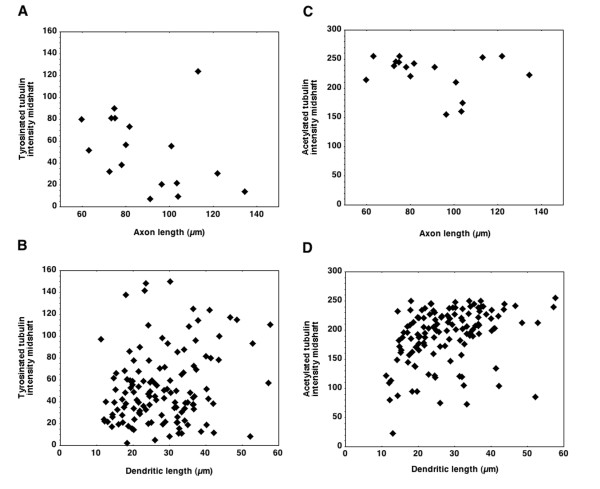
**Relationship between fluorescence intensity (in arbitrary units) of Tyr-tubulin and Ac-tubulin at the mid-shaft and process length in 2-DIV neurons**. **(A) **Intensity of Tyr-tubulin plotted against total axon length suggests an inverse relationship between length and amount of dynamic microtubules in the shaft, as predicted from previous work. **(B) **A plot of Tyr-tubulin and dendrite length does not show the same relationship. **(C, D) **Measures of Ac-tubulin in axons (C) and dendrites (D) are not suggestive of a linear relationship either. n = 16 neurons.

Previous analyses of microtubule stability in growing axons has led to the hypothesis that there is a predictable relationship between increased length and increased stability along the existing shaft [18,44]. If this is the case, then one might expect that with increased length, the intensity of staining for dynamic microtubules should decrease within the shaft. To test this prediction, the fluorescence intensity of Tyr-tubulin was measured at mid-shaft and plotted against process length for both axons and dendrites of neurons at 2 DIV (Figure 4). This prediction was supported by measures of dynamic microtubules in axons, but not in dendrites. The longer the axon, the weaker the intensity of dynamic Tyr-tubulin staining in the middle segment of the axon (Figure 4A). There was no evidence for a similar inverse relationship between length and dynamic Tyr-tubulin fluorescence in dendrites (Figure 4B).

**Figure 5:**
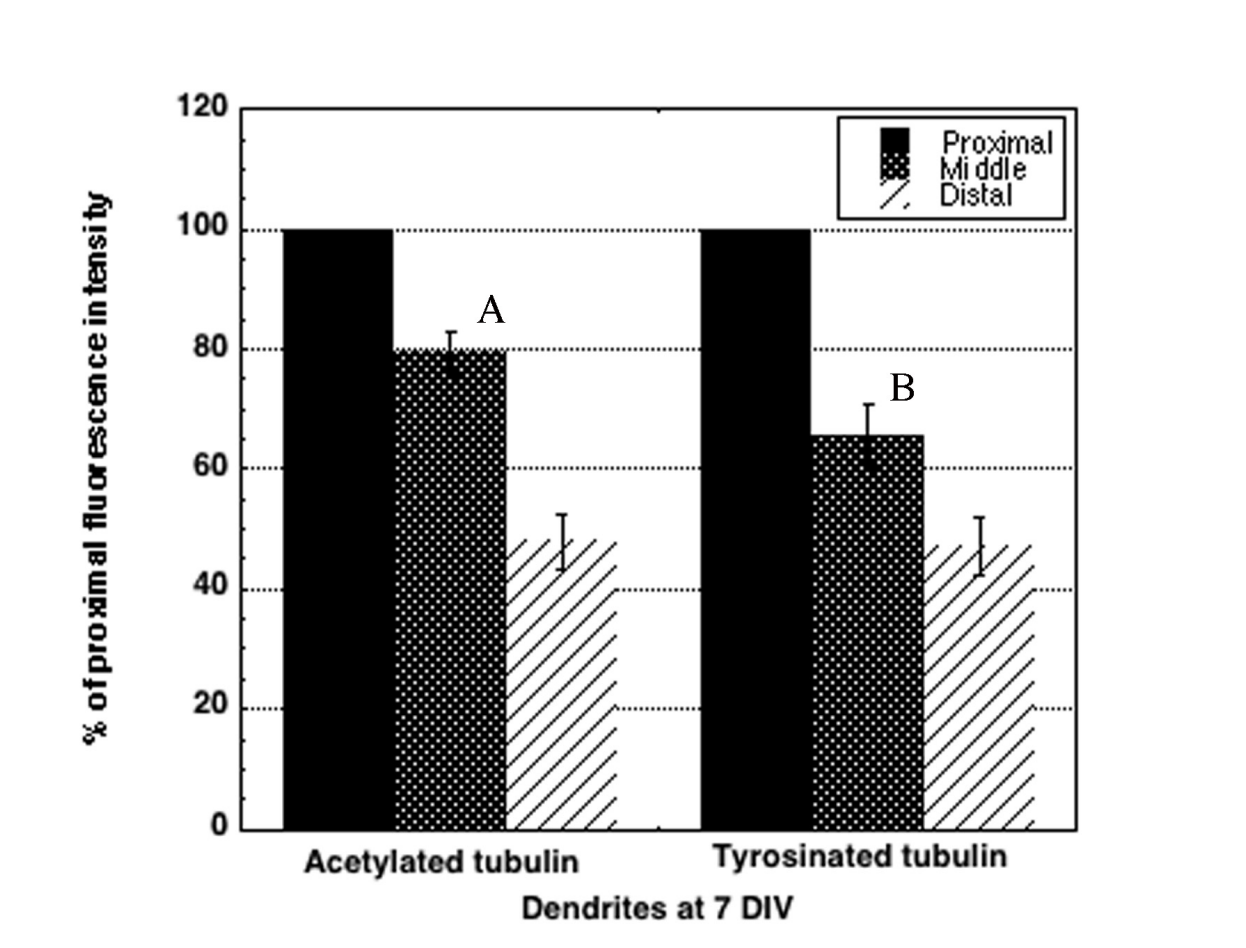
**Dynamic microtubules remain high in dendrites at 7 DIV**. Comparison of immunofluorescence intensity in the mid- and distal dendritic shaft of neurons at 7 DIV shows both Ac-tubulin and Tyr-tubulin decline as the dendrites taper. At the mid-shaft, Tyr-tubulin is about 65% of its original intensity at the origination of the dendrite from the soma, whereas Ac-tubulin has decreased to 78%. These values are significantly different from each other (distinguished by different letters, A versus B, *P *< 0.05). Both Ac and Tyr have decreased to about 48% of the original intensity in distal regions (excluding the last 10 μm of distal tip/growth cone region). Error bars represent standard error of the mean.

A related question is whether the intensity of staining for stable microtubules within the shaft might increase with increased length. We did not find such a relationship; immunfluorescence of stable Ac-tubulin in the shaft did not appear to increase with axon length (Figure 4C), nor with increased length in dendrites (Figure 4D). Because dendrites mature more slowly than axons, it is possible that with increasing age, dendrites would more closely resemble axons in shaft stability, and levels of Tyr-tubulin would decrease. However, analysis of cultures at 7 DIV, when the dendritic arbor has formed and branches are tapered, supported the qualitative observations shown in Figure 2. Within the middle segment of dendrites, Tyr-tubulin intensity did not decrease as dendrites matured and became longer (correlation coefficient between length and fluorescence intensity at midshaft = 0.21, data not shown). In addition, quantitative analysis of fluorescence intensity at the midshaft and distal dendrites of a subset of dendrites that were clearly isolated at 7 DIV showed that, while Tyr-tubulin is slightly decreased compared to Ac-tubulin, immunofluorescence for Tyr-tubulin is still relatively high when compared as a percentage of initial fluorescence in the proximal-most region (Figure 5). Tyr-tubulin did not appear to decrease substantially within the dendritic shaft of mature neurons either (Figure 2J–L), but as axons became intertwined with dendrites, it was not possible to identify dendrites and axons in isolation in order to make the same quantitative measurements. Combined, all these measures suggest that dendrites maintain a greater pool of dynamic microtubules in proportion to stable ones throughout development than do axons, which may have implications for ongoing plasticity.

### Microtubule polymerization is not restricted to growing ends of axons and dendrites

**Figure 6:**
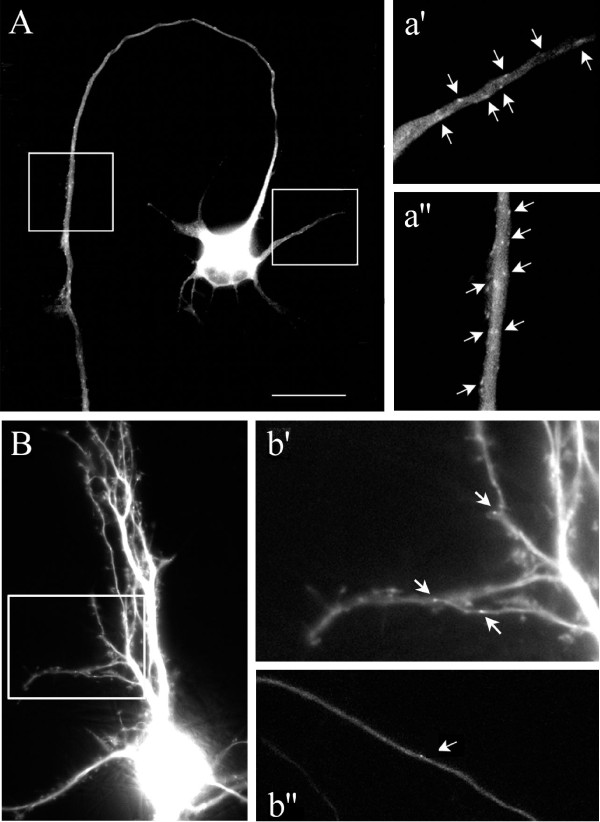
**EB1-GFP dynamics**. **(A-b") **Time-lapse imaging of EB1-GFP dynamics revealed diffuse low-level fluorescence and bright punctate comets (arrows) distributed throughout the axons and dendrites of hippocampal neurons developing *in vitro*. Representative still frames from movies, with higher magnification views of the boxed areas, of neurons at 1 DIV (A) (dendrite (a') and axon (a")) and 15 DIV (B) (dendrite (b') and axon (b")). Scale bar: 15 μm (A, B). See also accompanying Additional files 2 (1 DIV dendrite), 3 (1 DIV axon shaft), 4 (15 DIV dendritic arbor), 5 (15 DIV axon midshaft), and 6 (15 DIV distal axon and growth cone, not shown in figure).

Imaging of living cells transfected with EB1-GFP enables comparison of the spatial distribution of microtubule polymerization within the axonal and dendritic compartments of a single cell. To determine if polymerization occurs preferentially in regions of active growth, or differs between axons and dendrites during development, we recorded and analyzed EB1-GFP comets in polarized neurons at different stages of dendritic maturity. Live cell imaging of neurons expressing EB1-GFP at 1 to 17 DIV revealed bright comets along neurites (Figure 6). Regardless of age, polymerization was evident not only at the distal growing ends of axons and dendrites, but throughout the arbors of both immature (Figure 6A; Additional files 2 and 3) and mature (Figure 6B; Additional files 4, 5 and 6) neurons. The majority of comets traveled unidirectionally for short distances (up to 3 μm) before disappearing. Treatment with nocodozole (1 μM) to interfere with microtubule polymerization eliminated the appearance of EB1-GFP comets, leaving only diffuse fluorescence throughout the soma and neuronal processes (data not shown), similar to previous observations [30]. These findings support previous reports that EB1-GFP binds selectively to actively polymerizing microtubule plus-ends within both developing axons and dendrites, similar to endogenous EB1, thus providing a reliable marker for tracking physiological changes in microtubule dynamics. Moreover, these data also confirm that active growth of microtubules is not restricted to the growing tips of neuronal processes, but rather, occurs throughout the length of both axons and dendrites.

### Microtubule polymerization becomes biased toward distal regions with neuronal development

**Figure 7:**
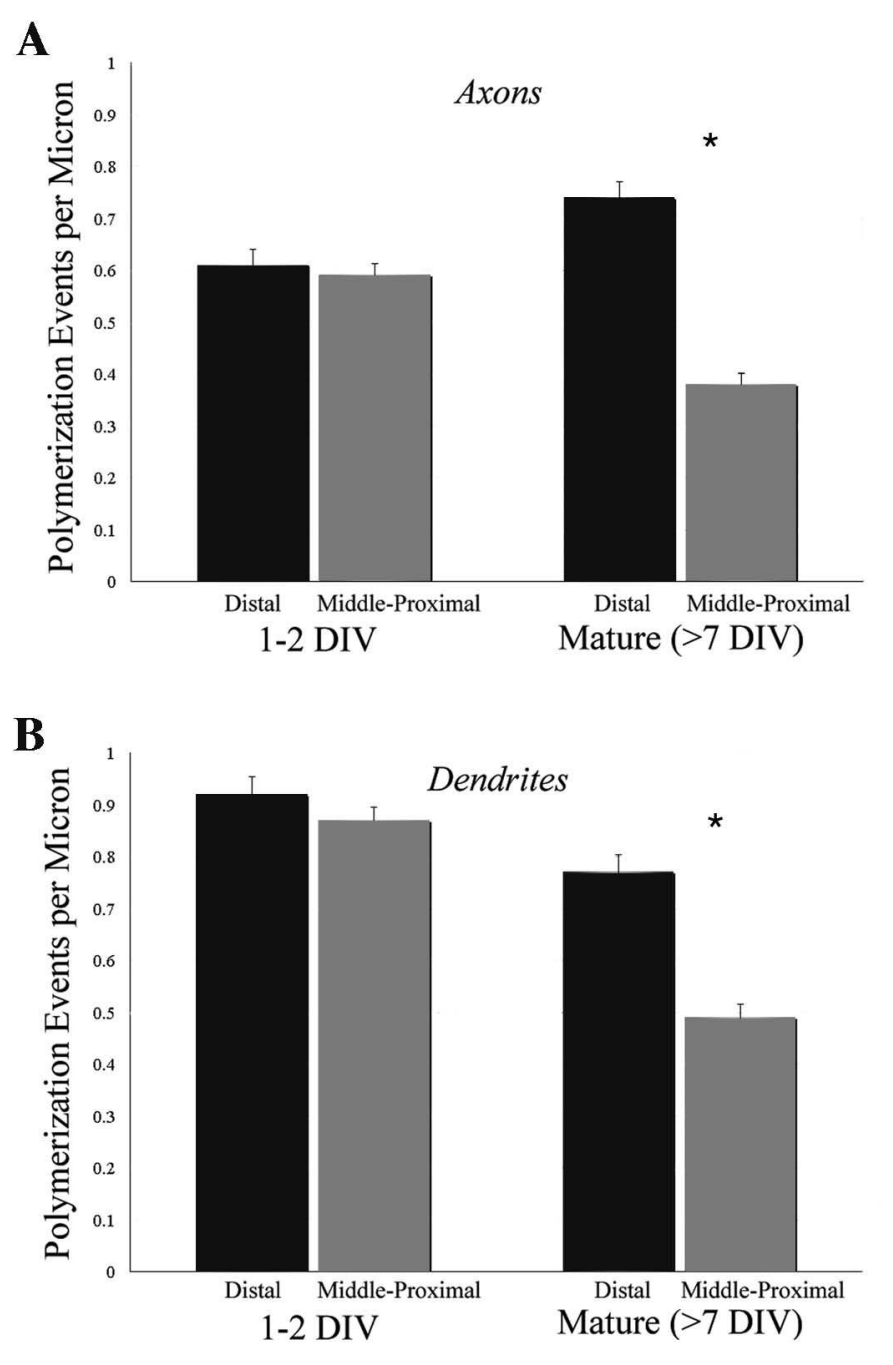
**The spatial distribution of microtubule polymerization becomes biased towards distal regions as axons and dendrites develop**. **(A, B) **The mean number of comets per micron was determined within segments representing the proximal-middle versus distal region (excluding the growth cone) of axons (A) and dendrites (B). For both axons and dendrites, microtubule polymerization was distributed equally within all regions at the earliest stages of development, but predominated within distal segment as neurons matured. Error bars represent standard error of the mean; *P *< 0.0001.

A longstanding hypothesis is that microtubule polymerization supports process outgrowth, and, thus, regions of active growth should show preferential polymerization even though lower level 'maintenance' polymerization might also persist throughout a process. If this is the case, then once an arbor is built, the number of polymerization events should decrease in stable regions, but might remain high (or increase) in distal regions that are still actively growing. To determine whether there was any regional bias in hippocampal neurons to changes in polymerization associated with development, we compared the spatial distribution of comets within the first 2 days in culture (1 to 2 DIV) with that of more mature neurons (7 to 17 DIV). Proximal-middle and distal (non-growth cone) regions of axons and dendrites were sampled randomly, and the number of fluorescent comets per unit length was determined (Figure 7).

At 1 to 2 DIV, comets were distributed equally across the proximal-middle and distal regions of both axons and dendrites. In more mature neurons (7 to 17 DIV), however, more polymerization events per unit length were observed in the distal regions of both axons and dendrites. These data suggest that with maturation, overall microtubule polymerization declines within the established portion of both the axonal and dendritic shafts, whereas distal regions of active growth maintain higher levels of polymerization, as predicted.

### The orientation of polymerizing microtubules changes with development

**Figure 8:**
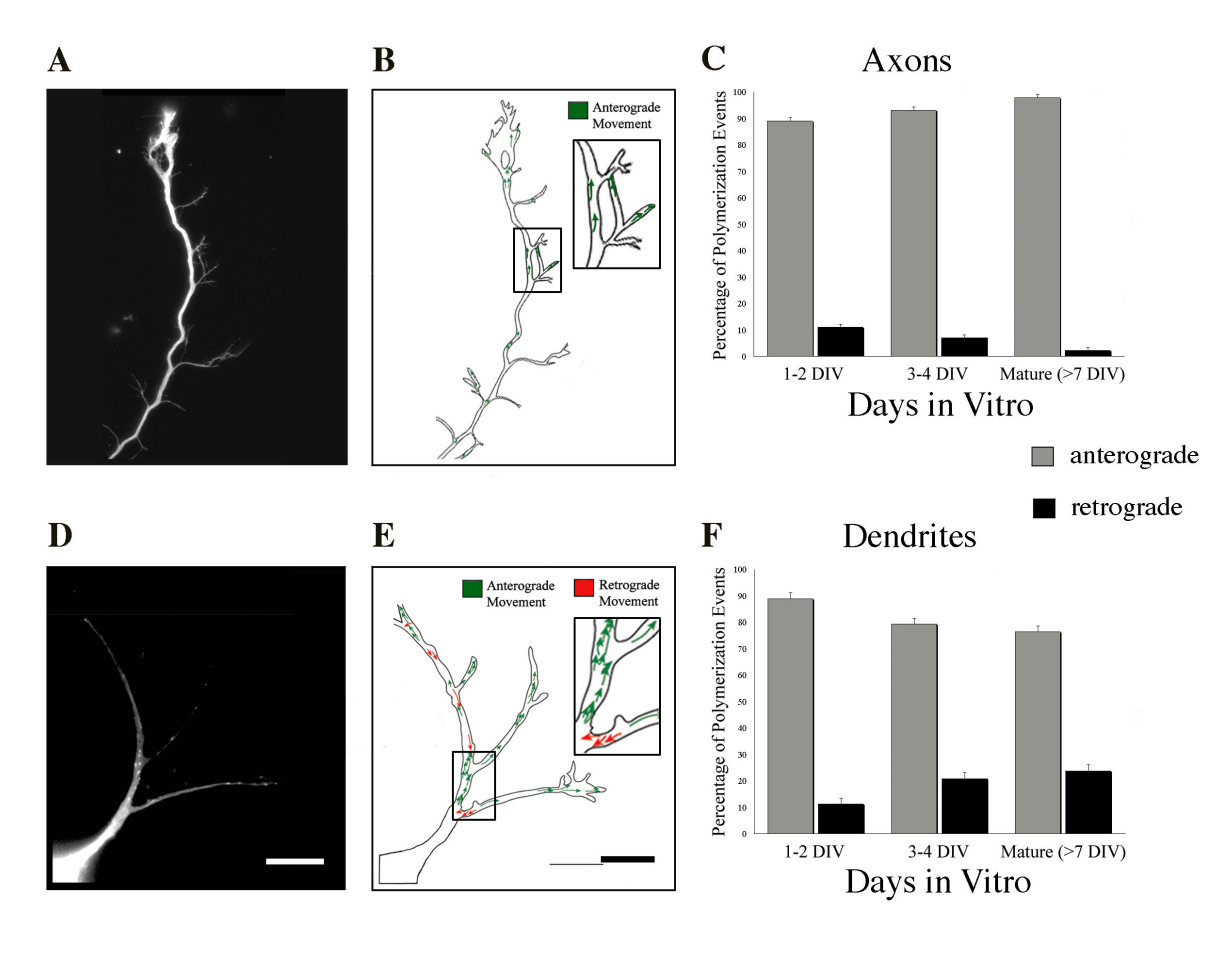
**As neurons mature, the proportion of microtubules polymerizing in the anterograde direction increases to nearly 100% in axons, whereas the number of retrogradely oriented events observed in dendrites increases**. **(A, D) **Single frames from the distal region of an axon (A) and a dendritic arbor (D) at 3 DIV, and **(B, E) **composite tracings of observed polymerization events, with individual EB1-GFP comet trajectories identified in the anterograde (green arrows) or retrograde (red arrows) directions. Insets depict enlarged views of the boxed regions. **(C, F) **The percentage of microtubule polymerization events occurring in an anterograde or retrograde direction was determined for each population of axons (C) and dendrites (F) observed throughout the developmental time-course. In axons, the total numbers of events tracked were: 833, 1 to 2 DIV; 892, 3–4 DIV; 284, 7+ DIV. For dendrites, the total numbers of events tracked were: 748, 1 to 2 DIV; 955, 3 to 4 DIV; 232, 7+ DIV. Scale bar, 10 μm. Error bars represent standard error of the mean. See Additional files 7 (axon) and 8 (dendritic arbor) for accompanying movies.

At the electron microscopic level following conditions allowing free-tubulin decoration, microtubules appear uniformly oriented with plus-ends distal within axons, but are oriented with both plus- and minus-ends distal in dendrites [24]. In the mature dendritic arbor, the proportion of microtubules with each polarity orientation is about equal. Such an analysis cannot reveal whether any of these microtubules are in a dynamic or stable state, and so leaves open the question of whether the directionality of active polymerization is proportional to the static distribution of microtubule polarity observed with the electron microscope. We tested the hypothesis that the overall directional pattern of polymerization would reflect the predicted distribution of microtubule polarity by quantifying the proportion of polymerization events that were anterograde (reflecting plus-end distal microtubules) and retrograde (reflecting minus-end distal microtubules) within developing axons and dendrites (Figure 8). In these analyses, we found that the relative proportion of anterograde and retrograde directed comets differed as a function of the ongoing development of axons and dendrites (Figure 8C, F; Additional files 7 and 8).

During the first 2 days in culture there was no difference in the overall directionality of microtubule polymerization between immature axons and dendrites of EB1-expressing polarized neurons, with most polymerization events occurring anterogradely. Approximately 11% of all comets traveled in the retrograde direction, a somewhat larger percentage than would have been predicted based on electron microscopy analyses of microtubule orientation (3 to 6%) [45]. As neurons matured, axons exhibited polymerization events that became nearly uniformly oriented in the anterograde direction, with fewer than 2% of the comets traveling in the retrograde direction (Figure 8C). Within dendrites, the proportion of retrograde events increased with time in culture (Figure 8F) as expected, but only reached 24% at the most advanced time-points measured (7 to 17 DIV) rather than the 50% expected. Taken together, these findings indicate that the directionality of microtubule polymerization mirrors the predicted distribution of anterogradely oriented microtubules in mature axons closely. Even with an increasing proportion of retrograde polymerization events, however, dendrites may have separate mechanisms controlling the gradual attainment of the characteristic mixed microtubule polarity and the distribution of microtubule polymerization.

### Analysis of rate and comparison with a neuron-like cell line, PC12 cells

Despite changes in the spatial distribution of EB1-GFP comets throughout development, the rates of polymerization did not differ between anterograde and retrograde events, nor did they differ between axons and dendrites (Table 1). Within axons, the rates of anterograde-directed microtubule polymerization ranged from 0.23 ± 0.02 μm/s at 1 to 2 DIV to 0.33 ± 0.02 μm/s at maturity. For dendrites, anterograde polymerization ranged from 0.25 ± 0.02 μm/s at 1 to 2 DIV to 0.22 ± 0.03 μm/s at maturity. The small number of retrograde events noted in axons after 3 DIV precluded statistical analysis of rates at maturity. In contrast, dendrites displayed increasing numbers of retrograde-directed EB1-GFP comets over the course of development, and the corresponding retrograde polymerization rates were similar with maturation. There is clearly variability in the observed rates. When considered together, however, similar anterograde and retrograde microtubule polymerization rates observed for axons and dendrites over development suggest that the kinetics of tubulin assembly are not affected by maturational changes in hippocampal neurons; moreover, these rates are in agreement with previously published rates in neurons [27,28,30].

**Table 1:** Microtubule polymerization rates in developing neurons and PC12 cells

Cell population	ANTEROGRADE EVENTS Mean Velocity (μm/sec ± SE)		RETROGRADE EVENTS Mean Velocity (μm/sec ± SE)	
Hippocampal neurons	Axons	Dendrites	Axons	Dendrites
1–2 DIV	0.23 ± 0.02 (142)	0.25 ± 0.02 (114)	0.29 ± 0.05 (22)	0.20 ± 0.05 (9)
3–4 DIV	0.26 ± 0.01 (214)	0.36 ± 0.02 (199)	0.43 ± 0.20 (5)	0.27 ± 0.03 (44)
> 7 DIV	0.33 ± 0.02 (86)	0.22 ± 0.03 (68)	0.35 (1)	0.18 ± 0.03 (21)
PC12 Cells	0.18 ± 0.003 (382)		0.17 ± 0.01 (86)	

For comparison with another cell type that extends processes, we measured the rate and orientation of polymerization events in PC12 cells after they had been induced to take on a neuron-like phenotype. Although these cells extend processes, they do not differentiate into axons or dendrites, but instead have features characteristic of each. Similar to neurons, microtubule polymerization in PC12 cells was evident throughout the length of the neurites (Additional file 9). Quantification of the number of EB1-GFP comets showed that while 82% of the polymerization events occurred in the anterograde direction, 18% were oriented in the retrograde direction (Table 1), suggesting similarity to mature dendrites rather than to axons in this measure.

## Discussion

Our findings suggest that even before the dendrites of cultured hippocampal neurons began to show signs of maturation (2 DIV), they contained a higher proportion of dynamic Tyr-tubulin throughout their arbor than did axons, and these high levels of Tyr-tubulin were maintained for several weeks. From these data, it appears that differential control over stability not only contributes to axon formation during the initiation of neuron polarity [6], but it also continues to distinguish dendrites from axons into maturity as well. The directionality of microtubule polymerization within axons and dendrites diverged later in development (3 to 4 DIV), with an increased incidence of retrogradely oriented polymerization that is closely aligned with the time-course of dendrite morphogenesis. This lag supports the hypothesis that changes in the orientation of microtubule polarity do not contribute to the initial specification of polarity, but may be important for later stages of dendritic maturation. Further strengthening this argument, interactions between retrogradely oriented microtubules and motor proteins, particularly dynein, have recently been shown to play a significant role in the transport and localization of dendritic proteins and organelles in mammalian models [35], as well as dendritic growth and branching in *Drosophila *neurons [46].

### Post-translational modifications of microtubules and the development of neuronal polarity

High levels of dynamic microtubules were present throughout the shaft of dendrites during development, as well as in mature neurons. This clearly contrasted with the distribution of microtubules in axons, where microtubules in the shafts were predominantly stable, whereas the microtubules in the distal ends were more dynamic, similar to axons of peripheral neurons [18-21]. These results fit well with previous reports of differential vulnerability to microtubule depolymerizing drugs between axons and dendrites of sympathetic neurons [20,47]; microtubules in the proximal and middle shaft of axons showed resistance to nocodozole treatment, whereas microtubules in dendrites were more vulnerable. More recent evidence suggests that interfering with post-translational modifications (for example, acetylation and detyrosination [36]) that are associated with microtubule stability can have significant effects on branching in axons [48] and cause aberrant outgrowth [49], potentially impacting the association of some +TIPs with microtubule ends [50]. Post-translational modifications of tubulin also contribute to the regulation of differential subcellular transport, in particular by altering the affinity of some transport vesicles and motors for particular microtubules [51,52]. Thus, local asymmetric increases in microtubule acetylation or detyrosination could bias kinesin-mediated transport to particular neurites, generating positive feedback signaling to allow for continued outgrowth and differentiation.

Subcellular changes in microtubule stability or transport could help to explain the different patterns of outgrowth that have been observed in different domains of cultured hippocampal neurons. While both axons and dendrites elongate and add collateral branches along their arbor, they can be distinguished by the rates of growth and patterns of motility once the axon becomes specified [9,10]. The axon extends rapidly, growing as much as several hundred microns per day, whereas the net growth rate of dendrites is very slow. Individual dendritic branches increase in length by about 10 μm/day and show frequent bouts of retraction as well as extension. A higher proportion of immature, and thus less stable, tubulin polymers within the shaft could contribute to the frequent retractions, and slow net growth of dendrites. Likewise, biased delivery of kinesin-associated vesicles to the more acetylated microtubules of the axon could also facilitate its rapid growth.

Considered together, the present data and earlier findings demonstrate that from the earliest specification of polarity and continuing into maturity, the axonal shaft is composed of relatively stable microtubules, whereas the dendritic arbor maintains a greater pool of dynamic microtubules. This is likely to reflect different requirements for dynamic instability that arise between the axonal and dendritic compartments, presumably important for establishing their distinct physiologies as neurons mature.

### Microtubule polymerization and neuron growth

Imaging of EB1-GFP comets revealed that microtubule polymerization occurs throughout the length of hippocampal neuron axons and dendrites, rather than being restricted solely to regions of active growth, such as axonal growth cones and dendritic tips. As these neurons continued to grow *in vitro*, however, the number of polymerization events declined within the older established portion of both axonal and dendritic shafts, whereas newer distal regions maintained higher levels of polymerization. Since distal ends constitute regions of active growth, such increased local polymerization suggests a shift toward ongoing assembly of the cytoskeleton on site, with lower level 'maintenance' polymerization along the shaft. Such a mechanism for regulating outgrowth in axons is well-supported (see, for example, [53]). The findings reported here suggest a similar mechanism for dendrites. Analyses of immunofluorescence showed concentrations of Tyr-tubulin at the distal tips of dendrites, and others have shown that regulation of tubulin polymerization within dendrite tips promotes dendritic growth and arborization [22,54]. Collectively, these data suggest a critical role for ongoing microtubule polymerization in dendritic arbors as well as axons as neuronal circuits are developing.

### Orientation of polymerization and differentiation of dendrites and axons

At the earliest time-points after hippocampal neurons became morphologically polarized, axons and dendrites could not be distinguished by directional patterns of microtubule polymerization. Rather, they began to diverge in the proportion of anterograde to retrograde polymerization events around 3 to 4 DIV. By 7 DIV, axons attained a nearly uniform distribution of anterograde polymerizing microtubules, while dendrites exhibited increased numbers of retrograde polymerization events. Retrograde polymerization had been detected in dendrites previously [30], a finding anticipated by previous electron microscopy studies [24,45]. What was unexpected, however, was that the directionality of microtubule polymerization in dendrites never reached roughly equal proportions of anterograde and retrograde events. Even neurons grown for more than 2 weeks *in vitro*, an age typically deemed mature by morphological and molecular criteria [1], continued to show a bias toward polymerization in the anterograde direction. One possible interpretation of this finding is that microtubules oriented with minus ends distal (thus polymerizing in the retrograde direction) are more stable than those with plus-ends distal (anterograde polymerizing). Regardless of the explanation for why the proportion of observed retrograde events is less than the expected total distribution of retrogradely oriented microtubules, these data suggest that the directionality of microtubule assembly is not simply proportional to the number of plus-end or minus-end distal microtubules present.

### Implications for regulation of growth and plasticity

Our findings show that a large pool of dynamic tyrosinated microtubules is available throughout the dendritic arbor even into maturity, a pattern that is distinctly different from that of axons. A population of dynamic microtubules could enable rapid cytoskeletal changes that support the growth of collateral branches from the dendrite shaft, a form of branching that occurs during outgrowth of the dendritic arbor in cultured hippocampal neurons (G Withers and G Banker, unpublished observations), and is likely to occur *in vivo *as well [55-57]. In addition, recent reports suggest that microtubule polymerization within the dendritic arbor, including transient forays into spines, could be important for spine formation, morphology, and activity-dependent changes in synapses [58-60]. Axons make collateral branches during pathfinding in development, but once the path is established, maintaining stability might be more important than retaining a capacity sprouting all along the axon tract. In contrast, it appears that the entire dendritic arbor is capable of growth, branch addition, and synapse formation throughout life. Thus, the differences in microtubule stability and dynamic polymerization between axons and dendrites reported here suggest a structural substrate that can account not only for differences in patterns of outgrowth, but also the capacity for plasticity.

## Conclusion

Significant differences in the organization of microtubules between dendrites and axons arise during development, but are maintained into maturation. Most notably, higher levels of dynamic tubulin, measured as Tyr-tubulin immunofluorescence, are retained throughout the dendritic arbor, whereas Tyr-tubulin is concentrated at distal growing ends of axons. While differences in microtubule stability can be detected even as neurons are polarizing, the proportion of retrograde to anterograde oriented polymerization diverges 1 to 2 days later, suggesting this aspect of microtubule organization may be more important for later phases of growth and maturation rather than in the initial specification of axons and dendrites.

## Abbreviations

Ac: acetylated; DIV: days *in vitro*; GFP: green fluorescent protein; Tyr: tyrosinated.

## Competing interests

The authors declare that they have no competing interests.

## Authors' contributions

Quantitative immunofluorescence experiments were done by RB and GW; imaging and analysis of EB1-transfected cells were conducted by KK, RB, MB and GW; KK, RB and GW contributed to the manuscript. All authors approved the final manuscript.

## Supplementary Material

Additional file 1 **Movement of EB-1 comets in a dendrite at 2 DIV**. Recording of a portion of a dendrite (at 2 DIV) from Figure 1 shows EB-1 comets traveling in both anterograde and retrograde directions (the accompanying kymograph is shown in Figure 1B, C). Movie duration, 10 s, with frames (500 ms exposure) acquired in a continuous stream. Click here for file

Additional file 2 **Movement of EB-1 comets in a dendrite at 1 DIV**. Segment of a dendrite (shown in Figure 6a') at 1 DIV showing most EB-1 comets traveling in the anterograde direction (toward the right), although retrograde events (toward the left) are clearly apparent as well. Click here for file

Additional file 3 **Movement of EB-1 comets in an axonal shaft at 1 DIV**. Segment of axonal shaft (shown in Figure 6a") at 1 DIV. Both anterograde (downward) and retrograde (upward) events are clearly evident in this segment, and could be detected throughout the entire axon length. Click here for file

Additional file 4 **Movement of EB-1 comets in a dendritic arbor at 15 DIV**. Dendritic arbor from a cell at 15 DIV. EB-1 comets can be detected throughout the arbor, traveling in both retrograde (toward the left) and anterograde (toward the right) directions, as well as entering a portion of dendritic spines. Click here for file

Additional file 5 **Movement of EB-1 comets in an axonal arbor at 15 DIV**. Anterogradely oriented EB-1 comets could also be observed throughout the axonal arbor at 15 DIV, although with less frequency than was evident in younger cells. This recording was taken from the middle region of an axon, with the cell body to the right, and growth cone to the left. Click here for file

Additional file 6 **Recording from the distal region and growth cone at 15 DIV**. Recording from the distal region, and growth cone (from the same cell as in Additional file 5) at 15 DIV. Click here for file

Additional file 7 **Movement of EB-1 comets in an axon at 3 DIV**. Accompanying movie of the 3-DIV axon shown in Figure 8A, B. Anterograde events are evident throughout the shaft of the axon, as well as the branches and filopodia that arise from the shaft. Click here for file

Additional file 8 **Movement of EB-1 comets in a dendritic arbor at 3 DIV**. Accompanying movie of the 3-DIV dendritic arbor shown in Figure 8D, E. Both anterograde and retrograde events throughout the length of the arbor are shown. Click here for file

Additional file 9 **Recording from a PC12 cell differentiated to take on a neuron-like phenotype**. Both anterograde and retrograde events were observed in these cells, in about the same proportions as were observed in the dendritic arbors of more mature neurons. Click here for file
